# Age-related Epstein-Barr virus-positive cutaneous ulcer arising after a self-limited subcutaneous abscess: a case report

**DOI:** 10.1186/1752-1947-6-288

**Published:** 2012-09-11

**Authors:** Shemsedin Sadiku, Fisnik Kurshumliu, Xhevdet Krasniqi, Ahmet Brovina, Emrush Kryeziu, Ibrahim Rrudhani, Kastriot Meqa, Lumturije Gashi-Luci, Hartmut Merz

**Affiliations:** 1 Hematology Clinic, University Clinical Center of Kosovo, “Rrethi i Spitalit” p.n,10000, Prishtina, Republic of Kosova; 2 Institute of Anatomic Pathology, University Clinical Center of Kosovo, “Rrethi i Spitalit” p.n,10000, Prishtina, Republic of Kosova; 3 Nephrology Clinic, University Clinical Center of Kosovo, “Rrethi i Spitalit” p.n,10000, Prishtina, Republic of Kosova; 4 Department of Periodontology and Oral Medicine, School of Dentistry, “Rrethi i Spitalit” p.n,10000, Prishtina, Republic of Kosova; 5 Institute of Pathology, University Clinic Schleswig-Holstein, Campus Luebeck, Germany; 6 German Reference Center, Campus Luebeck, Germany Consultation Centre for Hematopathology and Lymphoproliferative Diseases, University Clinic Schleswig-Holstein, Campus Luebeck, Germany

## Abstract

**Introduction:**

Epstein-Barr virus-positive mucocutaneous ulcer is a newly recognized clinicopathologic entity in the spectrum of Epstein-Barr virus-positive lymphoproliferative disorders. This entity is characterized by a self-limited, indolent course.

**Case presentation:**

We report the case of a 74-year-old, type 2 diabetic man who presented with an ulceroinfiltrative skin lesion on the left side of his neck. Histological examination showed that the lesion consisted of large atypical cells, some with Hodgkin-Reed-Sternberg-like morphology, in the midst of reactive lymphocytes, plasma cells, eosinophils and histiocytes. The atypical cells were partially positive for CD45, CD20, CD79a, CD30, B-cell lymphoma 2 and latent membrane protein 1 (CS.1-4), and negative for CD15, B-cell lymphoma 6 and CD10. *In situ* hybridization for Epstein-Barr virus-encoded ribonucleic acid was positive. Two years before, the patient had been diagnosed with a self-limited subcutaneous abscess in the same anatomic area that healed after antibiotic therapy.

**Conclusion:**

Older patients with positive Epstein-Barr virus serology may develop B-cell lymphoproliferations due to age-related immune suppression. Epstein-Barr virus-encoded ribonucleic acid testing and clonality analysis, eventually complemented with close clinical follow-up, should be performed for suspicious inflammatory lesions in older patients.

## Introduction

Epstein-Barr virus (EBV)-positive mucocutaneous ulcer is a newly recognized clinicopathologic entity in the spectrum of EBV-positive lymphoproliferative disorders (EBV-LPD) with a self-limited, indolent course [1]. It is associated with various forms of immunosuppression, including immunosenescence [1,2]. The localized nature of the disease may be due to a minimal and transitory gap in T-cell immunosurveillance [1-5].

By adulthood, more than 90% of individuals have become infected with EBV due to oral transmission [1-14]. The viral envelope glycoprotein, gp350, has the capacity to bind to the corresponding receptor on B lymphocytes, the CD21 molecule. After insertion, the B cells become persistently infected with the virus and may become transformed and proliferate indefinitely if T cell immunity is compromised [1-5]. It is well established that T cells play a major role in controlling EBV-associated oncogenesis [1-5]. This is complemented by the fact that administration of immunosuppressive agents after transplantation may lead to EBV-LPD [1-7].

## Case presentation

**Figure 1:**
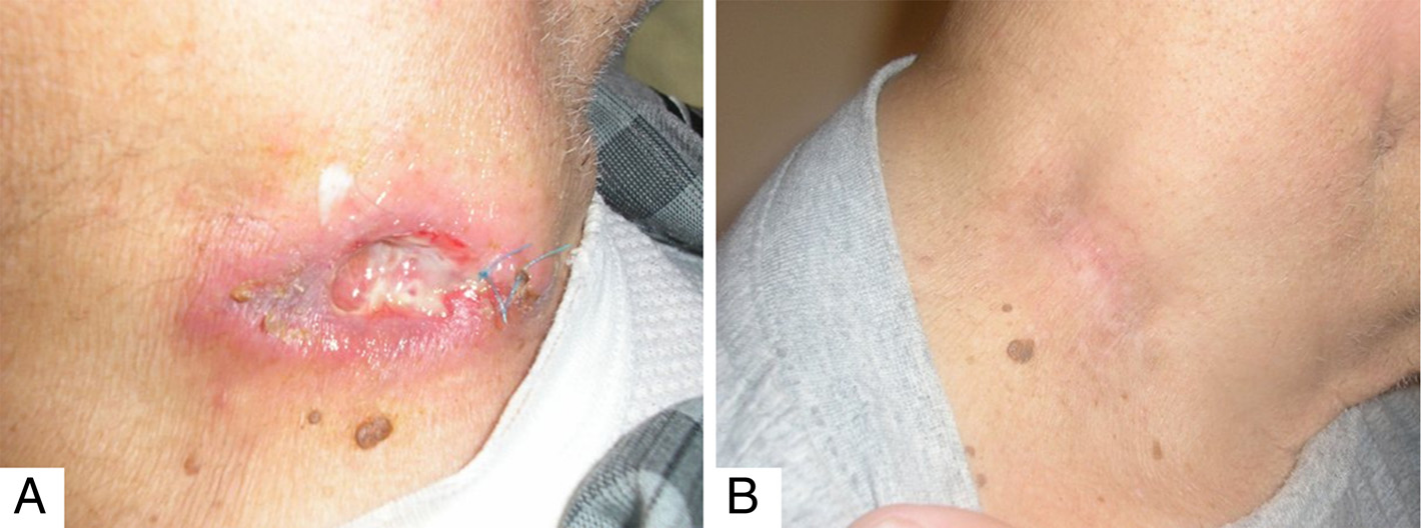
Gross appearance of the neck lesion at presentation (A) and after cyclophosphamide, doxorubicin, vincristine, prednisone, and rituximab (CHOP + R) therapy (B).

A 74-year-old type 2 diabetic man presented with a persistent ulceroinfiltrative skin lesion in the neck (Figure 1A).

Two years before, the patient had had a self-limited subcutaneous non-specific absceding inflammation in the same site which healed after antibiotic therapy. Examination of a blood smear revealed slight hypochromic anemia and unremarkable white blood cells and platelets. The serum lactate dehydrogenase (LDH) was normal. Subsequent computed tomography (CT) of the chest and ultrasound of the abdomen were unremarkable.

**Figure 2:**
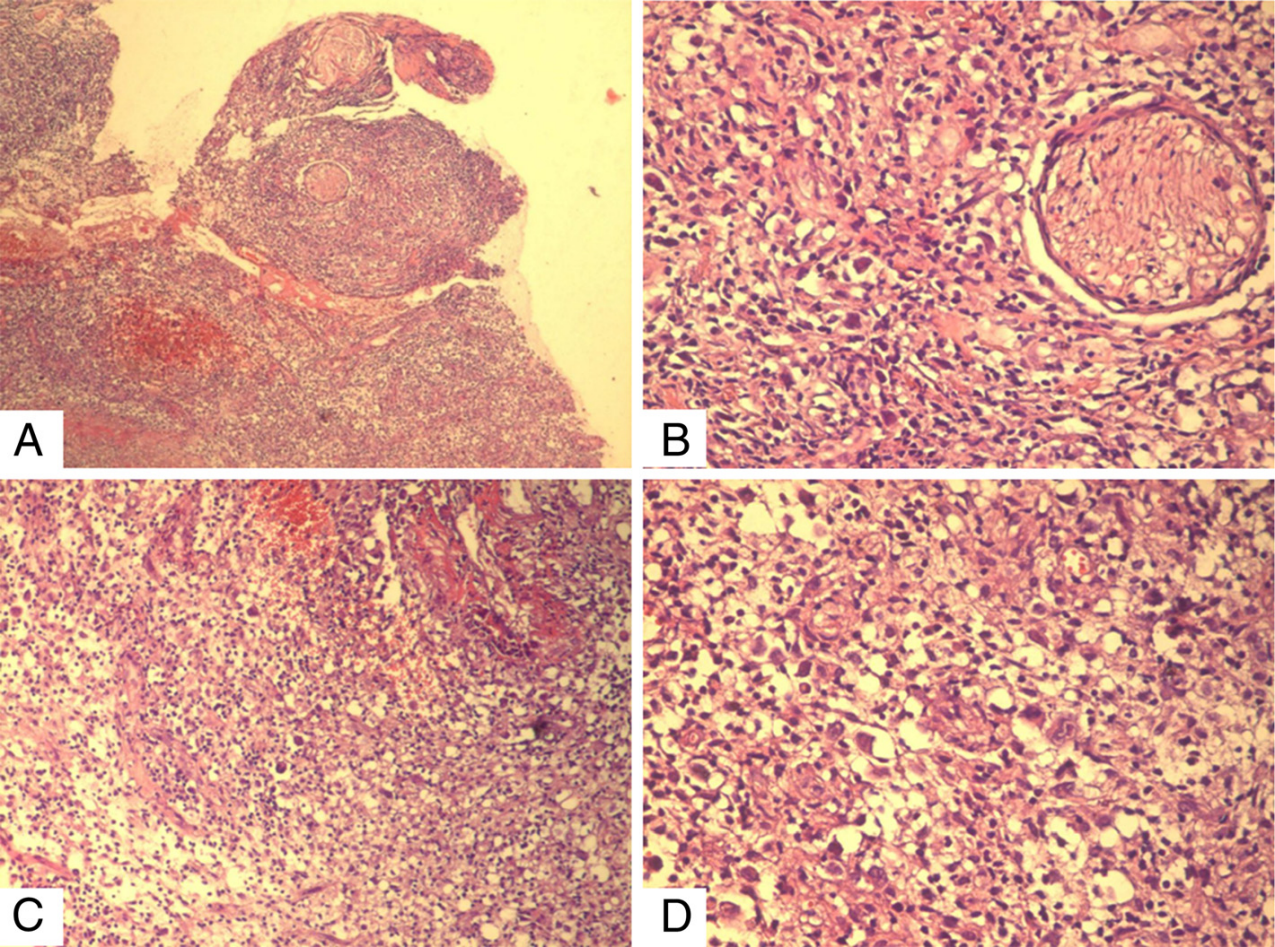
**Microscopic features of the (A) polymorphic lymphoid infiltrate extending between striated muscle cells and surrounding adnexal epidermal structures (×5; H&E stain) and (B) peripheral nerves (×20; H&E stain).** (**C**) There are confluent areas of necrosis and hemorrhage (×10; H&E stain). (**D**) In the midst of small reactive cells are large atypical Reed-Sternberg-like tumor cells (×20; H&E stain). H & E, haematoxylin and eosin.

Histopathology examination revealed the presence of large atypical cells, some with Hodgkin-Reed-Sternberg (HRS)-like morphology, in the midst of reactive lymphocytes, plasma cells, eosinophils and histiocytes (Figures 2A-D).

**Figure 3:**
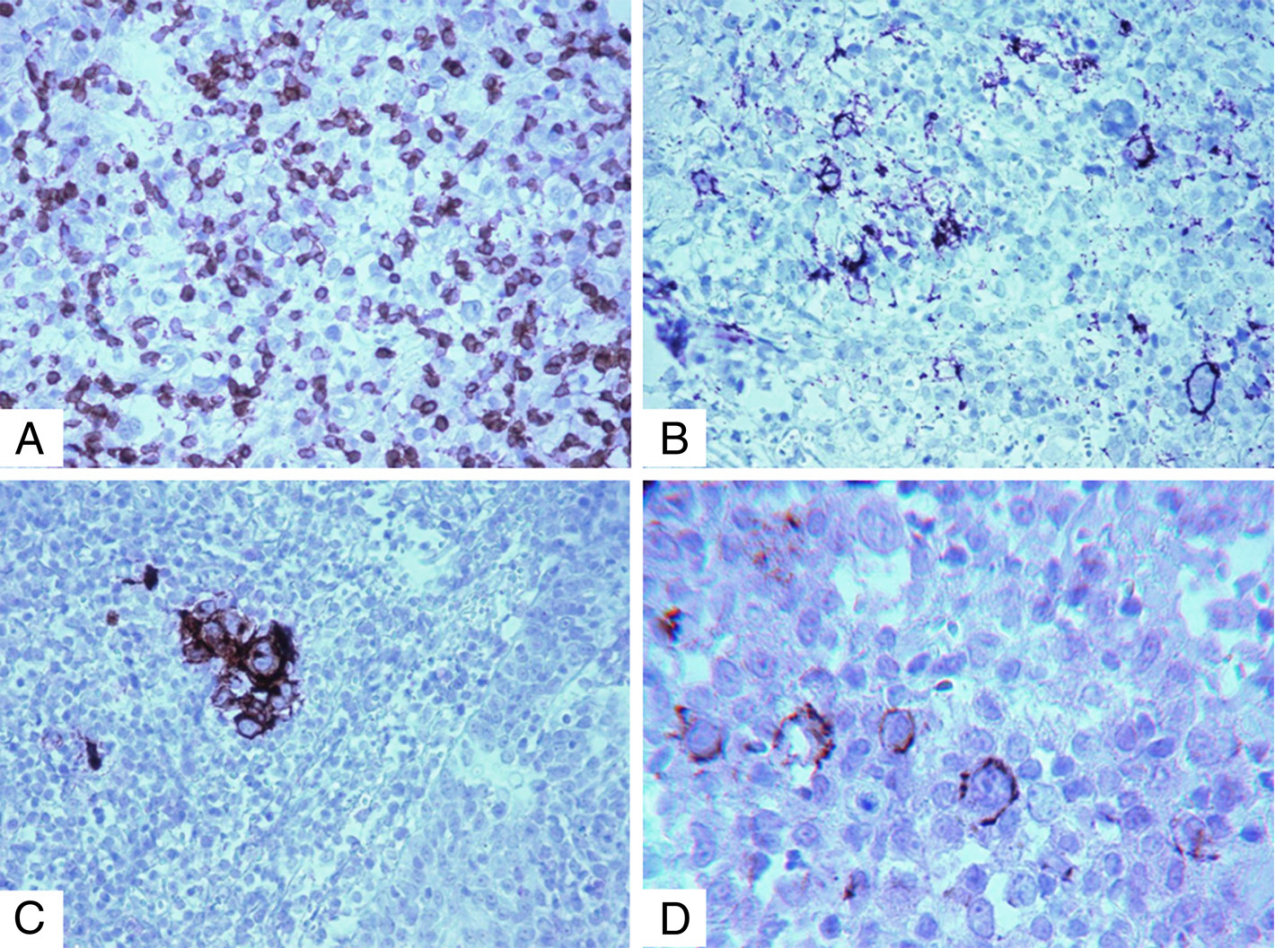
Immunohistochemical analysis illustrates (A) negative reaction of the neoplastic cells for CD3 (×10; immunoperoxidase stain), (B) positive reaction for CD20 (×10; immunoperoxidase stain), (C) positive reaction for CD30 (×10; immunoperoxidase stain) and (D) Latent membrane protein 1 of Epstein-Barr virus (×20; immunoperoxidase stain).

The atypical cells were partially positive for CD45, CD20, CD79a, CD30, B-cell lymphoma 2 (Bcl-2) and latent membrane protein 1 (LMP1) (CS.1-4), while negative for CD15, B-cell lymphoma 6 (Bcl-6) and CD10. The surrounding cells were CD3+/CD4+/CD8+/CD20+ reactive T and B cells associated with many CD68+ histiocytes (Figures 3A-D). No immunoglobulin light chain restriction could be demonstrated in paraffin-embedded tissue immunohistochemistry. There were confluent areas of “geographic” necrosis. The infiltrate extended in between striated muscle and around peripheral nerves.

**Figure 4:**
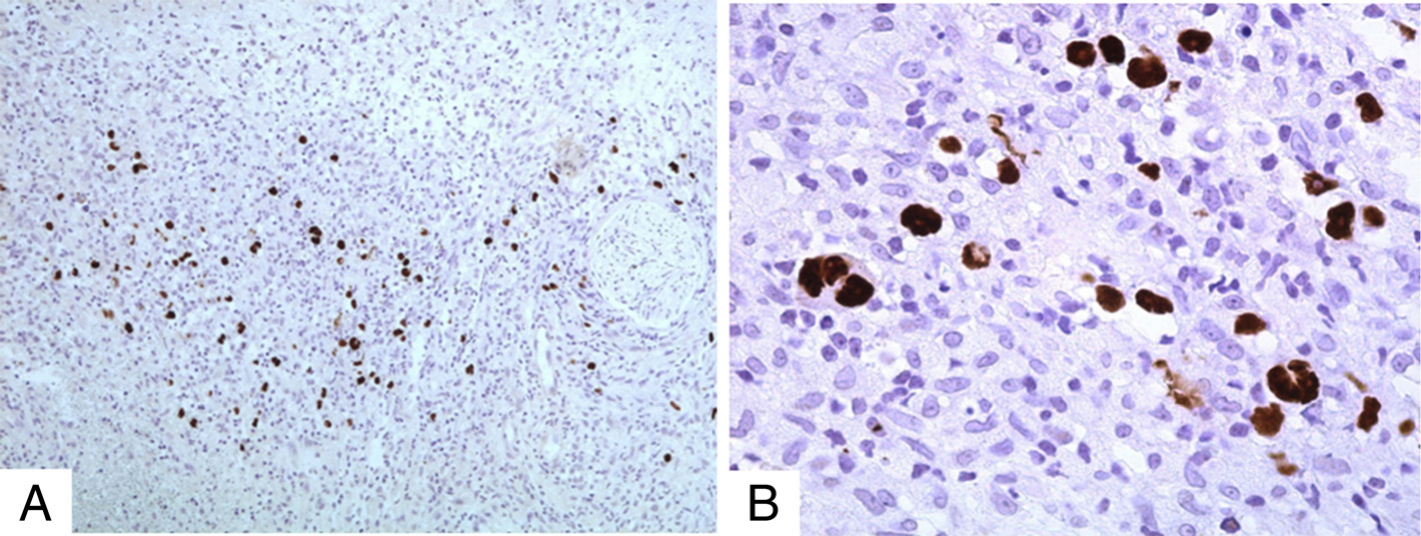
**Epstein-Barr virus-encoded ribonucleic acid** ***in situ*** **hybridization illustrates (A and B) positive nuclear reaction of the large atypical and Reed-Sternberg-like cells (×10 and x20; chromogenic** ***in situ*** **hybridization stain).**

The large atypical cells were positive for Epstein-Barr virus-encoded RNA (EBER) *in situ* hybridization (Figure 4A and B).

He underwent treatment with a combination chemotherapy regimen consisting of cyclophosphamide, doxorubicin, vincristine, prednisone, and rituximab (CHOP + R) that resulted in complete remission (Figure 1B).

He remains well after a follow-up period of two years.

## Discussion

EBV-positive mucocutaneous ulcer (EBVMCU) is a recently described EBV positive B-cell lymphoproliferation that occurs in patients >50 years old due to iatrogenic or age related immunosuppression [1,2,10,14]. This entity takes part in the spectrum of EBV-associated lymphoproliferative disorders together with reactive lymph node hyperplasia, polymorphic nodal lymphoproliferative disease and diffuse large B-cell lymphoma [1,2]. Two studies by Dojcinov *et al*. [1,2] characterize this lesion as a shallow, sharply circumscribed mucosal or cutaneous ulcer with a histological picture of large pleomorphic blasts reminiscent of HRS cells associated with variable numbers of reactive lymphocytes, plasma cells, histiocytes and eosinophils [1,2]. Besides histological features described by the author, our case displays a deeper lesion with prominent perineural invasion and infiltration between striated muscle. In spite of the deeper extension of this lesion, the clinical presentation of our case is more in line with EBVMCU than the EBV-positive diffuse large B-cell lymphoma of the elderly which is characterized by an aggressive clinical course [1,2,10,14].

As described in the literature**,** we, too, consider that the border between reactive lesions and lymphoma can be imprecise and classification between different types of age related EBV lymphoproliferative disorder (AR-EBVLPD) subtypes can be problematic [2].

EBV is causally related to a number of lymphoid neoplasms, such as Burkitt lymphoma, extranodal NK/T-cell lymphoma nasal type, angioimmunoblastic T-cell lymphoma, some cases of classical Hodgkin’s lymphoma (cHL), lymphomatoid granulomatosis, plasmablastic lymphoma, primary effusion lymphoma and diffuse large B-cell lymphoma associated with chronic inflammation [10,14]. On the other hand, there is a spectrum of lymphoproliferative EBV-related lesions ranging from infectious mononucleosis, a non-neoplastic self-limited infectious syndrome to EBV-positive lymphoproliferative disease of childhood, a potentially fatal lymphoproliferation [10,14]. Chronic active EBV infection is considered as an entity that lies in between these two different prognostic categories, with a potential of evolving to a clonal and aggressive lymphoproliferation [10].

Our case has many overlapping features with lymphomatoid granulomatosis (LyG) and cHL. In more than 90% of cases, LyG presents with bilateral lung involvement which was not the case in our patient [10,14]. The presence of HRS-like cells in the background of inflammatory cells raises the possibility of cHL [10,14]. However, it is highly unusual for cHL to present at an extranodal location [1,2,10,14]. Determination of immunophenotype of the tumor cells is very helpful given that HRS cells are usually positive both for CD30 and CD15 and may variably and heterogeneously express CD20 [10,14]. In the vast majority of cases of diffuse large B-cell lymphoma (DLBCL) expressing CD30, the neoplastic cells are CD15 negative [10,14].

Nonetheless, separation between DLBCL and cHL is becoming more and more difficult in some cases despite the ever advancing diagnostic means. The emergence of the entity designated as B-cell lymphoma unclassifiable, with features intermediate between DLBCL and cHL is in line with this conclusion [14]. Also, according to the emerging literature, in EBV-LPD, including DLBCL, the tumor cells express CD15 in concert with CD30 in a number of cases [1,2,10,14]. It is well established that cHL is a neoplasm of germinal center B-cell derivation due to the demonstration of weak Pax-5 expression by HRS cells by immunohistochemistry [10,14]. In retrospect, two years ago, our patient had presented with a left-sided cervical mass that in histology was revealed to be non-specific chronic absceding inflammation. Until the current clinical presentation the patient had had an uneventful clinical history, besides the underlying diabetes.

## Conclusions

The fact that the current lesion was located in the same anatomical area as the previously diagnosed abscess raises the theoretical possibility of a stepwise process that gradually evolved from a reactive process to oligoclonal and clonal neoplastic proliferation. Another hypothesis is the possibility of the first lesion to be an overlooked EBV-LPD that underwent spontaneous regression, given that EBER *in situ* hybridization and clonality analysis were not performed.

“Non-specific” inflammatory lesions of unusual locations in older patients should be viewed with caution due to the possibility of synchronous or metachronous neoplasia. When in the slightest doubt, EBER testing and clonality analysis should be performed, eventually complemented with close clinical follow-up.

## Consent

Written informed consent was obtained from the patient for publication of this case report and any accompanying images. A copy of the written consent is available for review by the Editor-in-Chief of this journal.

## Competing interests

The authors declare that they have no competing interests.

## Authors’ contributions

ShS analyzed, interpreted and provided the patient data. FK reviewed the slides and wrote the manuscript. XhK analyzed and interpreted patient data and contributed to the manuscript. AB analyzed patient data. EK analyzed patient data. IR analyzed patient data. KM provided clinical data and the clinical pictures. LGL analyzed patient data. HM was a major consultant for slide review, performed the EBER stain and provided the relevant articles. All authors read and approved the final manuscript.
